# Implications for a Deeper Clinical Understanding of Moyamoya Disease: Temporal Evolution of Suzuki Staging

**DOI:** 10.1227/neuprac.0000000000000256

**Published:** 2026-06-16

**Authors:** Joshua M. Cohen, Hailey D. Reisert, Joshua Stich, Serena Zhang, Ryan Fatemi, Margaret Keymakh, Geena Jung, Rutvi Patel, Genesis Liriano, Mandana Behbahani, Andrew J. Kobets

**Affiliations:** 1 Albert Einstein College of Medicine, Montefiore Medical Center, Bronx, New York, USA;; 2 Department of Pediatric Neurology, Montefiore Medical Center, Bronx, New York, USA;; 3 Department of Neurosurgery, Montefiore Medical Center, Bronx, New York, USA;; 4 Current affiliation: Memorial Sloan Kettering, New York, New York, USA

**Keywords:** Internal carotid artery stenosis, Moyamoya disease, Suzuki stage, Temporal progression, Vasculopathy

## Abstract

**BACKGROUND AND OBJECTIVES:**

Moyamoya disease (MMD) is a rare cerebrovascular disorder discovered in Japan, characterized by progressive internal carotid artery stenosis and fragile collateral vessel formation. Diagnosis relies on imaging, with Suzuki staging commonly used to classify disease severity. There is limited literature on MMD in non-Asian, non-White populations. This study evaluated clinical factors associated with disease presentation, severity, treatment, and progression, with a novel focus on longitudinal changes in Suzuki stage.

**METHODS:**

A retrospective cohort study was conducted using electronic medical records. Clinical data, including demographics, Suzuki stages, surgical interventions, stroke frequency, and outcomes, were collected and analyzed.

**RESULTS:**

Of 114 patients diagnosed with MMD, 89 were from underrepresented populations in Moyamoya literature. Imaging from 91 patients showed Suzuki scores following a bell-curve distribution. Higher initial Suzuki scores were significantly associated with an increased likelihood of surgery (*P* < .001). In a subcohort of 71 patients with at least 2 Suzuki scores 1 year apart, scores remained largely stable, although a weak, nonsignificant trend toward worsening was seen in nonsurgical patients (*P* = .14). Notably, Mann-Whitney *U* analysis revealed a significant difference in Suzuki stage changes, with nonsurgical patients showing greater radiographic progression and surgically treated patients exhibiting stability or improvement (*P* < .05). No significant correlation was found between changes in Suzuki stage and functional outcomes or stroke events.

**CONCLUSION:**

This study expands understanding of MMD in a diverse, non-Asian population and supports Suzuki staging as a reliable tool for disease assessment and surgical planning. Surgical intervention appears to slow radiographic progression, supporting its role in management. Further prospective studies are needed to refine Suzuki staging in monitoring disease trajectory and guiding treatment.

**ABBREVIATIONS:**
EDAS: encephaloduroarteriosynangiosis
MMD: moyamoya disease
MRA: magnetic resonance angiography
NIR: near-infrared spectroscopy
SCD: sickle cell disease.

First described by Suzuki and Takaku in 1957, moyamoya disease (MMD) is characterized by progressive stenosis of the distal internal carotid arteries (ICAs) and the formation of fragile, compensatory collateral vessels at the skull base. These collateral vessels, which develop in the early stages of MMD, represent the brain's attempt to augment blood flow in a hypoperfused state. Patients with a unilateral or bilateral circle of Willis occlusion are diagnosed with MMD.^1^ By contrast, patients presenting with the characteristic vascular occlusion alongside recognized conditions such as sickle cell disease (SCD) or neurofibromatosis type 1 are classified as having moyamoya syndrome.^1^ Moyamoya syndrome was originally hypothesized to have a different pathophysiology than MMD, although recent studies have found no significant clinical or radiological differences in presentation.^2^

## Diagnosis

MMD is diagnosed using imaging criteria, with magnetic resonance angiography (MRA) or computed tomography angiography (CTA) preferred for their noninvasive evaluation of the ICA and associated vasculature. Diagnostic criteria include ICA stenosis, irregular vasculature, and bilateral findings in definitive cases.^1,3^ Pathological features including thickening of the circle of Willis and small branching vessels aid in diagnosis.^3,4^

## Suzuki Grade and Angiographic Findings

- Stage I: Minimal narrowing of the distal ICA near the carotid fork, with relatively preserved blood flow and no significant collateral vessel formation.
- Stage II: The beginning of collateral formation, where increased stenosis of the ICA triggers the appearance of small, fragile vessels (“moyamoya vessels”).
- Stage III: Intensified stenosis, leading to a greater density and complexity of moyamoya vessels, resulting in the characteristic “puff of smoke” angiographic appearance.
- Stage IV: The density of moyamoya vessels starts to decline as the ICA becomes further occluded, and compensatory collateral circulation from the external carotid artery (ECA) may begin to develop to sustain cerebral perfusion.
- Stage V: Moyamoya vessels further decrease as the occlusion advances, with blood flow increasingly dependent on ECA collaterals.
- Stage VI: Complete occlusion of the ICA and the disappearance of most moyamoya vessels, leaving cerebral perfusion almost entirely reliant on ECA collaterals.^4^

## Treatments

Surgical revascularization is an effective treatment for restoring sufficient collateral circulation in MMD patients with cerebral ischemia. Postoperative benefits include improved cerebral hemodynamics, reduced frequency and severity of ischemic events, and favorable long-term neurological outcomes.^5^ Revascularization techniques are categorized as direct, such as superficial temporal artery-to-middle cerebral artery anastomosis, and indirect, such as encephaloduroarteriosynangiosis (EDAS). These methods can be used alone or in combination, with the choice of procedure influenced by factors such as surgeon preference, age group, and individual anatomic differences.^6,7^

Despite Suzuki grading being the oldest documented method of recording moyamoya progression, treatment decisions and directive cases for Suzuki grade remain largely subjective and vary widely between institutions. This study aimed to evaluate moyamoya demographics and Suzuki grades in a single tertiary care center as well as reevaluate the utility of Suzuki grading as a reliable and applicable diagnostic tool. By retrospectively analyzing MRA, CTA, and near-infrared spectroscopy (NIR), we assessed Suzuki grades at multiple time points and investigated the relationship between Suzuki grade, clinical interventions, and patient outcomes.

## METHODS

### Study Design

The study was a retrospective cohort analysis to evaluate the demographic characteristics, indications for treatment modalities, diagnostic criteria, and outcomes of patients with MMD. In addition, multiple Suzuki grades were assessed for patients with more than 1 scan at least 1 year apart to evaluate the natural history and progression of the disease. Data were collected from the electronic medical record system at Montefiore Medical Center from July 2011 to December 2022. This study adhered to Strengthening the Reporting of Observational Studies in Epidemiology guidelines for reporting cohort studies.

Institutional review board approval was obtained from the Montefiore Medical Center Institutional Review Board. Given the retrospective nature of the study and use of de-identified data, the requirement for informed patient consent was waived by the institutional review board.

### Population and Sample

Patients were identified using International Classification of Diseases, 10th revision codes for MMD (I67.5) in the electronic medical record system. Inclusion criteria included MMD confirmed by imaging studies and complete data on demographic information. A total of 114 patients met the inclusion criteria.

### Data Collection

The patient cohort was characterized by demographics (age, gender, comorbidities), presenting symptoms, stroke history, and treatments. Patients were classified using Suzuki grading. Authors J.M.C. and R.F. independently reviewed imaging; interpretations were concordant for 83 patients, with the remaining 8 interpreted by R.P. to minimize bias. ICA stenosis was categorized as mild/moderate (<70%), “stenosis severity score 1,” severe (71%-99%, “2”), or complete occlusion (no detectable flow, “3”). Imaging modalities for diagnosis and Suzuki grading included MRA, CTA, and NIR. Data on surgical revascularization and outcomes were collected. The original Glasgow Outcome Scale (GOS) was used to evaluate functional outcomes, scored from 1 to 5 (1 = death, 2 = persistent vegetative state, 3 = severe disability, 4 = moderate disability, and 5 = good recovery). Patients with additional imaging were assigned a second Suzuki score, and the years between the 2 imaging sequences were recorded as years of follow-up.

### Data Analysis

All analyses were performed in GraphPad Prism version 10.3.1 (GraphPad Software). A *P*-value <.05 was considered statistically significant. Continuous variables were compared using Kruskal-Wallis tests (nonparametric one-way analysis of variance) with multiple comparison correct using the two-stage step-up method of Benjamini, Krieger, and Yekutieli. Comparisons between 2 independent non-normally distributed groups used the Mann-Whitney *U* test (Wilcoxon rank-sum test). Categorical variables were analyzed using χ^2^ or Fisher exact tests. Spearman correlations were computed.

## RESULTS

### Participant Demographics

Between 2011 and 2022, 114 patients were diagnosed with moyamoya with available demographic data (Table 1). Most were adults (n = 73, 64%), with a mean age at diagnosis of 29 years (range 1-71), and were female (n = 83, 73%). Spanish/Hispanic/Latino patients were predominantly adults, whereas most Black or African American patients were pediatric; White and Asian patients had equal adult and pediatric representation (*P* = .0005). SCD was the most common comorbidity (n = 35, 31%), consistent with prior literature.^1,7^

**TABLE 1.** Demographics for Entire Cohort

Demographics n (%)			
	Total	Pediatrics	Adult
Total cases	114	41 (36.0)	73 (64.0)
Sex			
Male	31 (27.2)	19 (61.3)	12 (38.7)
Female	83 (72.8)	22 (53.7)	61 (83.6)
Race			
Spanish/Hispanic/Latino	51 (44.7)	11 (21.6)	40 (78.4)
White	4 (3.5)	2 (50)	2 (50)
Black or African American	38 (33.3)	23 (60.5)	15 (39.5)
Asian	4 (3.5)	2 (50)	2 (50)
Other/unknown	9 (7.9)	1 (11.1)	8 (88.9)
Declined	8 (7.0)	2 (25)	6 (75)
Comorbidities			
Sickle cell disease	35 (30.7)	29 (82.8)	6 (17.2)
Sickle cell trait	2 (1.8)	0 (0)	2 (100)
Down syndrome	2 (1.8)	1 (50)	1 (50)
Chronic meningitis	1 (0.8)	0 (0)	1 (100)
Brain/skull base tumor	2 (1.8)	0 (0)	2 (100)
Thyroid disease	8 (7.0)	1 (12.5)	7 (87.5)
Cerebral vasculitis	2 (1.8)	0 (0)	2 (100)
Autoimmune disease	6 (5.3)	0 (0)	6 (100)

### Presenting Symptoms

Eighty-five patients were diagnosed with MMD after a stroke (76%), which were further categorized as ischemic (n = 75) or hemorrhagic (n = 8). The second most common presentation was moyamoya recognized as an incidental finding during evaluation for SCD (n = 18, 16%). In the pediatric population, the SCD incidental diagnosis was more common, with over one-third of patients being diagnosed in this manner (n = 15, 37%). In the adult population, stroke was the most common reason for MMD diagnosis (n = 63, 86%). Other presenting symptoms at diagnosis are presented in Table 2.

**TABLE 2.** Presenting Symptoms

Presenting symptoms entire cohort (n = 114)		
Ischemic stroke (%)	75 (66)	
Hemorrhagic stroke (%)	8 (7)	
SCD (incidental finding) (%)	18 (16)	
Other (%)		
Headache	2 (1.8)	11 (9.6)
Stroke-like symptoms	1 (0.9)	
Rheumatic fever	1 (0.9)	
ICP	1 (0.9)	
Unknown	6 (5.3)	

	Pediatric (n = 41)	Adult (n = 73)	*P*-value
Presenting symptom by age group (%)			
Stroke	22 (54)	63 (86)	<.0001
SCD	15 (37)	3 (4)	
Other	4 (10)	7 (10)	

	Spanish/Hispanic/Latino (n = 51)	Black/African American (n = 38)	Other/unknown/declined (n = 25)	*P*-value
Presenting symptom by racial group (%)				
Stroke	42 (82)	24 (63)	19 (76)	.03
SCD	7 (14)	10 (26)	1 (4)	
Other	2 (4)	4 (11)	5 (20)	

ICP, intracranial pressure; SCD, sickle cell disease.

Presenting symptoms were then evaluated by age and racial groups. Analyses were performed with all stroke types combined. Stroke was the most common presenting symptom for MMD in all racial groups, followed by SCD and then other causes. SCD was the presenting symptom for MMD in 10 (26%) of Black or African American patients, significantly higher than that of the other groups (Table 2). Hemorrhagic stroke was associated with a significantly lower median GOS than all 3 other presentation groups (median difference 1.0; 95% CI 0-2 vs ischemic, 1-2 vs SCD, 0-2 vs other/unknown; *P* = .001).

### Procedure Characteristics

**FIGURE 1.**
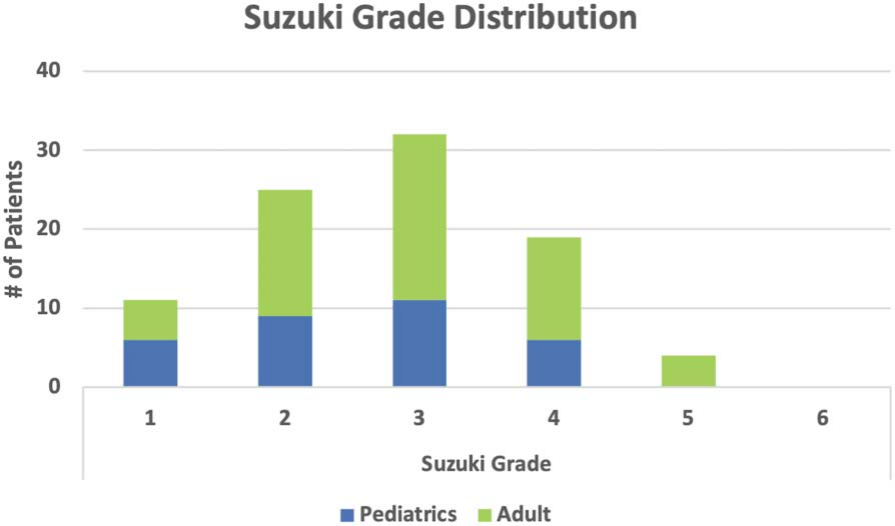
Initial Suzuki grade distribution.

Of 114 patients, 91 received imaging at diagnosis with MRA, CTA, or NIR, allowing for further characterization of MMD. Suzuki scores followed a bell-curve distribution with the highest number of patients receiving a grade of 3 (n = 32) and the lowest number of patients receiving a 5 and 6 (n = 3 and 0, respectively; Figure 1). Severe stenosis (70%-99% occlusion) was the most common degree of ICA occlusion at the time of diagnosis (n = 46, 51%) with complete occlusion (100%) as the second most common (n = 28, 31%).

A total of 41 patients received surgical treatment for moyamoya (46%), with 37 receiving EDAS, 1 receiving a superficial temporal artery-middle cerebral artery anastomosis, and 3 receiving both surgical options. The average time from diagnosis to surgery was 16.8 months (range 0-120 months). Postoperatively, 4 patients experienced a stroke (10%) within 4 months of surgery, while 25 patients saw marked improvement in blood flow to the circle of Willis (61%).

### Associations Between Initial Suzuki Grade and Clinical Factors

There were no statistically significant differences in initial Suzuki score by patient sex, age, or age group (i.e., pediatric vs adult; Table 3). There was a significant positive association between the initial Suzuki score and ICA stenosis severity, with a greater degree of occlusion being present in higher Suzuki scores (*P* < .001). A majority of the cohort presented with bilateral findings (n = 110, 96.5%), and data from only the more severely affected side were included in the analysis.

**TABLE 3.** Initial Suzuki Score

Variable	Suzuki score					*P*-value
	1	2	3	4	5*P*-value	
Sex (%)						
Male	3 (27)	7 (27)	8 (26)	5 (26)	1 (25)	>.99
Female	8 (73)	19 (73)	23 (74)	14 (74)	3 (75)	
Age						
Median (SD)	17 (14.3)	29 (16.8)	34 (18.3)	35 (19.9)	48 (17.8)	.39
Age group (%)						
Pediatrics	6 (55)	10 (38)	10 (32)	6 (32)	0 (0)	.42
Adult	5 (45)	16 (62)	21 (68)	13 (68)	4 (100)	
ICA stenosis (%)						
1 (<70% occlusion)	6 (55)	9 (35)	2 (7)	0 (0)	0 (0)	**.0008**
2 (70%-99% occlusion)	3 (27)	13 (50)	18 (60)	10 (53)	1 (25)	
3 (100% occlusion)	2 (18)	4 (15)	10 (33)	9 (47)	3 (75)	
Management (%)						
Surgical	1 (9)	7 (27)	16 (52)	13 (68)	4 (100)	**.0005**
Nonsurgical	10 (91)	19 (73)	15 (48)	6 (32)	0 (0)	

ICA, internal carotid artery.

Percentages within each variable equal fraction of column total. Analyses were χ^2^. Bold entrance indicate *P*-value < .05.

There was a statistically significant association between the initial Suzuki score and surgical management, with higher scores receiving surgery at a greater percentage of the time (100% for Suzuki score of 5 vs 9%, 27%, 52%, 68% for Suzuki 1-4, respectively, *P* < .001; Table 3). Similarly, there was a statistically significant association between ICA stenosis severity and surgical management (65% of patients with complete occlusion had surgery, 45% for severe stenosis, 24% for mild/moderate stenosis, *P* < .05). The relationship between the initial Suzuki score and postoperative stroke events was not significant.

### Temporal Changes in Suzuki Grade During Follow-Up

A second Suzuki score was available for 71 patients with follow-up imaging >1 year after diagnosis (Table 4; median age 26 years, range 1-59). Of these, 42% were pediatric (<18 years) and 58% adult (≥18 years), and 32 of these 71 patients received surgical intervention (45%). The median follow-up was slightly shorter in adults (8 years) than pediatric patients (9 years). Among adults, surgically treated patients had shorter follow-up (7.5 vs 10 years), whereas among pediatric patients, surgically managed individuals had longer follow-up (10 vs 9 years).

**TABLE 4.** Demographics for Suzuki Change Cohort

Demographics n (%)			
	Total	Pediatrics	Adult
Total cases	71	30 (42)	41 (58)
Sex			
Male	19	16 (84)	3 (16)
Female	52	14 (27)	38 (73)
Median age at diagnosis (y)	26	10.5	40
Median length of follow-up (y)	9	9	8
Surgical intervention received	32	6 (19)	26 (81)

The length of time between diagnostic and most recent imaging for this cohort was median 5 years (range 1-15 years). To reiterate, N = 32 (45%) received surgical treatment during the follow-up period between the initial imaging and most recent imaging. Change in Suzuki score over the follow-up period ranged from −2 to +2 (median 0), denoted as Delta S. The number of stroke events over the follow-up period ranged from 0 to 7 (median 1).

**FIGURE 2.**
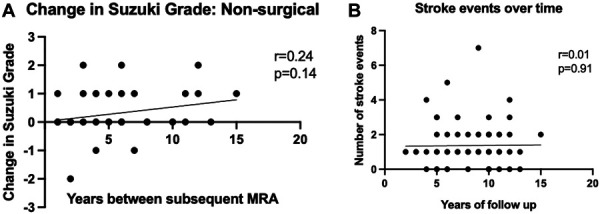
**A**, Number of stroke events over time. **B**, Change in Suzuki grade in nonsurgical patients over time. MRA, magnetic resonance angiography.

The duration of follow-up and the number of stroke events was not significant, r(72) = 0.01, *P* = .91 (Figure 2A). There was a nonsignificant weakly positive correlation between the number of years between imaging and change in Suzuki score, r(72) = 0.24, *P* = .14, such that Suzuki score increased (i.e., got worse) with time over the follow-up period (Figure 2B).

**FIGURE 3.**
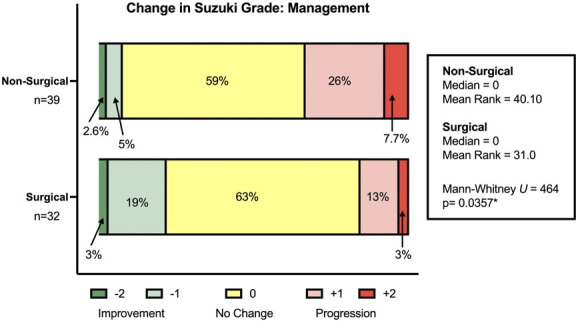
Mann-Whitney *U* Analysis of change in Suzuki grade by management.

Among the 71 patients with a second Suzuki score, change in Suzuki stage did not differ between pediatric patients compared with adult patients using a Mann-Whitney *U* test (Table 5). Change in Suzuki score between surgical and nonsurgical groups was compared using a Mann-Whitney *U* test. Although the median change in Suzuki stage was the same in both groups (median = 0), the distributions differed significantly (U = 464, *P* = .0186). The nonsurgical group demonstrated a higher, more positive distribution of Suzuki score change (mean rank = 40.10), indicating a greater proportion of patients with radiographic progression, whereas the surgical group demonstrated a lower, more negative distribution (mean rank = 31.0), reflecting more patients with stability or improvement in disease severity (Figure 3).

**TABLE 5.** Change in Suzuki Score Over Follow-up Period (n = 71)

		Delta S	Mann–Whitney *U*	*P*-value
Age group Mean,^a^ (SD)	Pediatrics	0 [0, 0]	512	.18
	Adults	0 [0, 1]		
Management Mean,^a^ (SD)	Surgical	0 [0, 0]	464	**.04**
	Nonsurgical	0 [0, 1]		
Age and management Mean,^b^ (SD)	Surgical pediatrics	−0.5 [−1.25, 0]	NA	**.01**
	Nonsurgical pediatrics	0 [0, 0.75]		
	Surgical adults	0 [0, 0]		
	Nonsurgical adults	0 [0, 1]		

NA, not applicable.

a Nonparametric Mann-Whitney *U* tests were used to compare group distributions for analyses of age group and management alone.

b Nonparametric Kruskal-Wallis test was used to compare age and management groups. All comparisons across groups were not statistically significant (*P* > .05).

Bold entries indicate *P*-value < .05.

**FIGURE 4.**
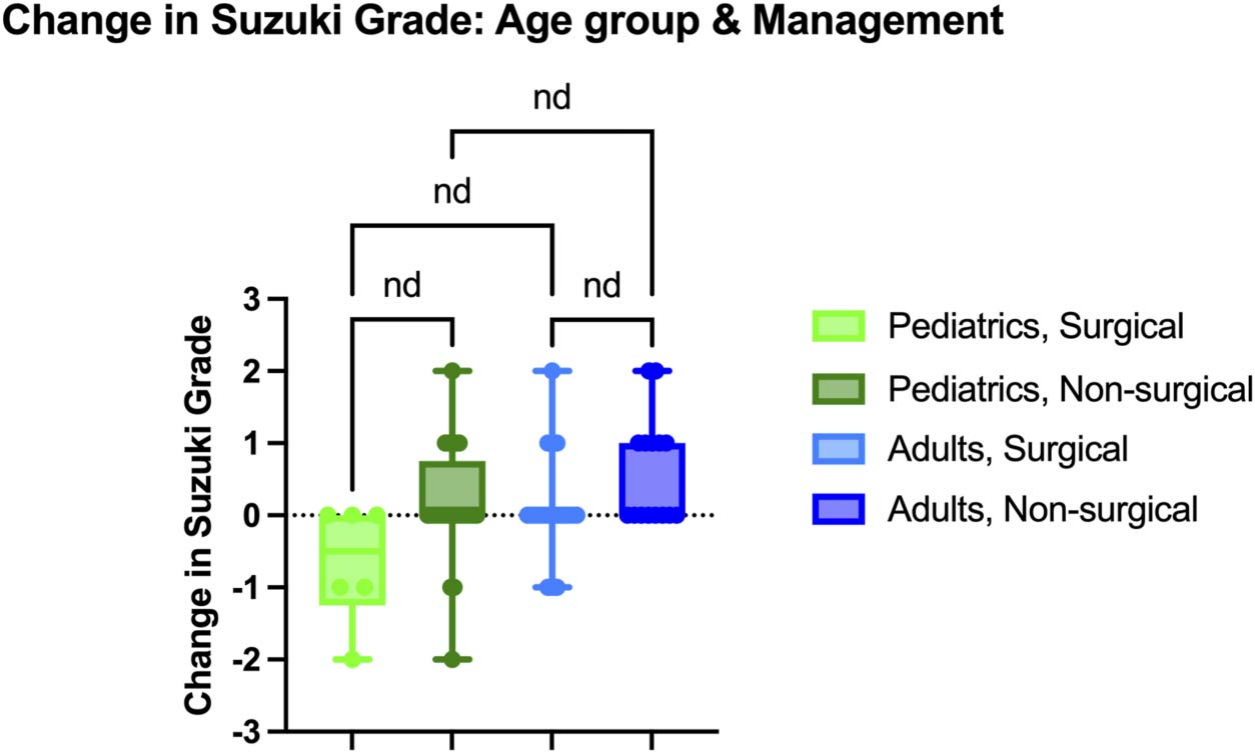
Change in Suzuki grade by age group and management.

When stratified by age and management group, no pairwise comparisons reached statistical significance, including surgically managed pediatric vs nonsurgically managed pediatric patients (*P* = .13), surgically managed pediatric vs surgically managed adults (*P* = .24), surgically managed adults vs nonsurgically managed adults (*P* = .13), and nonsurgically managed pediatric vs nonsurgically managed adults (*P* = .32; Figure 4).

We further evaluated the effect of surgical intervention on patients with SCD. Patients with SCD who underwent surgery experienced an improvement in Suzuki score compared with non-SCD patients who were managed nonsurgically (*P* < .05). A direct comparison of patients with SCD by surgical management was not significant (*P* = .07).

### Clinical Outcomes

Functional status at follow-up was assessed using the GOS. The median GOS score in both the surgical and nonsurgical groups was 5, the most favorable outcome. No statistically significant difference in GOS score was observed between the groups (*P* = .4355). Furthermore, there was no significant difference in GOS score by age group (*P* = .0833).

We did not observe a statistically significant correlation between the change in Suzuki score and number of stroke events over the follow-up period.

## DISCUSSION

MMD is a complex vasculopathy with unclear pathoetiology and management.^8^ Although MMD has been extensively described in East Asian populations and, more recently, in White populations, Hispanic and Black/African American populations remain underrepresented.^9-11^ Our study addresses this gap by examining a diverse cohort in which stroke was the most common presentation across groups. Notably, 26% of Black/African American patients had MMD as a manifestation of SCD, often diagnosed incidentally during routine SCD evaluation, highlighting an important consideration for physicians managing SCD.

Reliable MMD studies remain scarce due to inconsistent definitions and absence of standardized longitudinal methods.^8,12^ Among proposed grading systems, the Suzuki grading system is the most widely used angiographic method.^13,14^ We retrospectively observed Suzuki stages and treatment decisions and compared ICA stenosis severity to Suzuki stage. Our findings affirmed this relationship: higher degrees of stenosis correlated with more advanced Suzuki stages.

Robert et al reported that moderate to severe ICA stenosis strongly predicts surgical intervention, yet there is no consensus on the revascularization indications. This uncertainty has led to institution-specific practices, compounded by small sample sizes.^8,14,15^ Our analysis contributes to the literature supporting Suzuki staging by examining our institution's relationship between operative management of MMD and Suzuki staging.

In our cohort, patients with milder disease (Suzuki 1-2) were generally managed conservatively, half of those with stage 3 underwent surgical management, and most with stages 4 to 5 received surgical management. Although this study does not define surgical thresholds, it highlights Suzuki staging as a tool to augment clinical judgement in surgical candidacy and monitoring progression.

Rosi et al^16^ argued that Suzuki staging is not a reliable independent indicator of disease progression, citing nonsignificant findings in their analysis. In our cohort, we observed no statistically significant progression overall using sequential imaging. However, a trend toward progression was noted among nonsurgical patients, consistent with the expected natural history of untreated MMD. This may reflect the limited sample size and variable follow-up duration, as several patients had <3 years of imaging data. Prospective studies with standardized imaging intervals will better capture disease progression over time.

While Suzuki staging has been validated diagnostically, prior studies have not evaluated temporal progression using sequential imaging.^17,18^ In 71 patients with at least 2 imaging studies 1 year apart, patients who did not undergo surgery demonstrated a nonsignificant tendency toward radiographic progression over time, where significant findings would have been consistent with the expected natural progression of untreated MMD. The Mann-Whitney *U* test indicated that although both surgical and nonsurgical groups had the same median change in Suzuki grade, the distributions differed significantly. Nonsurgical patients showed a higher, more positive distribution of Suzuki score change, reflecting a greater proportion with disease progression, whereas surgically treated patients had a lower, more negative distribution, indicating more patients with stability or improvement.

**FIGURE 5.**
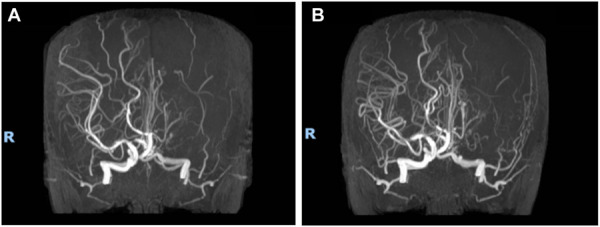
Preoperative and postoperative magnetic resonance angiography imaging for a pediatric patient with sickle cell disease: **A**, Preoperative, Suzuki grade 4. **B**, Postoperative, Suzuki 3.

Among patients with SCD, a significant difference between surgical SCD and nonsurgical non-SCD group demonstrates that surgery may be particularly effective in SCD-associated MMD. To illustrate this finding, we include preoperative and postoperative imaging from a pediatric SCD patient showing postoperative improvement in Suzuki grade (Figure 5). Although most subgroup comparisons were not significant, likely due to sample size, these trends highlight the potential impact of surgery on disease progression.

In patients undergoing EDAS, neovascularization following extracranial-to-intracranial bypass may alter ICA diameter and reflect both therapeutic effect and Suzuki grade improvement. The clinical implications of this vascular remodeling are nuanced and merit additional study. Larger multicenter studies will be necessary to further evaluate these trends across diverse patient populations.

To evaluate functional outcomes, we used the GOS, which provides a 1 to 5 rating of a patient's clinical status at the time of review. The median GOS score in both groups was 5, indicating a highly skewed distribution toward favorable outcomes, and no significant difference was observed between the surgical and nonsurgical groups. In addition, no statistically significant relationship was observed between length of follow-up and number of stroke events nor between surgical management and stroke frequency. The former analysis confirmed that longer follow-up did not account for increased stroke rates. Our finding that surgical management was not associated with stroke events contracted with prior literature, such as Gao et al, which reported lower stroke recurrence after revascularization.^15,16,19^

Future research should use prospective modalities to evaluate moyamoya progression and further validate Suzuki staging as a tool for diagnosis and treatment planning. Although Suzuki staging informed decision making in this study, it should complement and not replace clinical decision making. Distinguishing presenting symptoms and stroke subtypes may further refine treatment protocols. Continued investigation into disease progression may establish Suzuki staging as integral in improving patient outcomes and guiding management decisions.

### Limitations

Despite these findings, several limitations warrant consideration. First, the retrospective design and single-system sample limit generalizability to the broader US moyamoya population. Interpretation of Suzuki grade progression is constrained by inconsistent follow-up intervals and nonstandardized imaging timelines. The small number of both surgically managed patients and total stroke events further limit comparisons with larger studies. Nonetheless, these data contribute to understanding the disease's natural history. Further studies may benefit from focusing on stroke occurrences to better identify opportunities for earlier intervention.

## CONCLUSIONS

In our retrospective analysis, we expanded understanding of MMD in a diverse population, identifying patterns in presentation, Suzuki grade, treatment, and outcomes. Our findings demonstrate the Suzuki staging system as a reliable indicator of disease status and a practical tool for tracking progression. Patients with higher scores at diagnosis were more likely to undergo surgical management. Notably, Mann-Whitney *U* analysis of surgical management demonstrated a statistically significant difference in the distribution of Suzuki stage change (i.e., stability, progression, or improvement in disease). Nonsurgical patients showed greater radiographic progression, while surgically treated patients showed more stability or improvement. These results underscore the potential therapeutic benefit of surgery and reinforce the potential utility of Suzuki staging in monitoring disease progression over time and treatment decision making. Future prospective studies should further validate Suzuki staging's role in guiding management and improving outcomes.

## References

[1] Scott RM Smith ER. Moyamoya disease and moyamoya syndrome. N Engl J Med. 2009;360(12):1226-1237. 19297575 10.1056/NEJMra0804622

[2] Schwartzmann Y Spektor S Moscovici S Comparison between moyamoya disease and moyamoya syndrome in Israel. J Stroke Cerebrovasc Dis. 2024;33(4):107635. 38342272 10.1016/j.jstrokecerebrovasdis.2024.107635

[3] Fukui M. Guidelines for the diagnosis and treatment of spontaneous occlusion of the circle of Willis (“Moyamoya” disease). Research Committee on Spontaneous Occlusion of the Circle of Willis (Moyamoya Disease) of the Ministry of Health and Welfare, Japan. Clin Neurol Neurosurg. 1997;99(Suppl 2):S238-S240. 9409446

[4] Suzuki J Takaku A. Cerebrovascular “moyamoya” disease: disease showing abnormal net-like vessels in base of brain. Arch Neurol. 1969;20(3):288-299. 5775283 10.1001/archneur.1969.00480090076012

[5] Minematsu K Toyoda K Hirano T Guidelines for the intravenous application of recombinant tissue-type plasminogen activator (alteplase), the second edition, October 2012: a guideline from the Japan Stroke Society. J Stroke Cerebrovasc Dis. 2013;22(5):571-600. 23727456 10.1016/j.jstrokecerebrovasdis.2013.04.001

[6] Matsushima T Inoue T Suzuki SO Fujii K Fukui M Hasuo K. Surgical treatment of moyamoya disease in pediatric patients: comparison between the results of indirect and direct revascularization procedures. Neurosurgery. 1992;31(3):401-405. 1407421 10.1227/00006123-199209000-00003

[7] Robert AP Hanel RA Adelson PD Indications for cerebral revascularization for moyamoya syndrome in pediatric sickle cell disease determined by Delphi methodology. J Neurosurg Pediatr. 2024;34(4):402-413. 39029127 10.3171/2024.5.PEDS2426 PMC12887912

[8] Rifino N Hervè D Acerbi F Diagnosis and management of adult moyamoya angiopathy: an overview of guideline recommendations and identification of future research directions. Int J Stroke. 2025;20(5):512-523. 39425621 10.1177/17474930241297031 PMC12089666

[9] Unda SR Antoniazzi AM Fluss R Ethnic-associated phenotype variations in moyamoya cerebrovascular outcomes. Cerebrovasc Dis. 2023;52(5):519-525. 36566750 10.1159/000528055 PMC10627485

[10] Ott WP Bellamy S Onyali CB. Recurrent transient neurological deficits in a young Hispanic woman: a case review of moyamoya disease. Am J Case Rep. 2023;24:e940353. 37528569 10.12659/AJCR.940353 PMC10405348

[11] Nathal E Serrano-Rubio A Maciel E Arauz A. Moyamoya disease in Mexico: our experience. Neurología (Engl Ed). 2021;36(8):603-610. 34654535 10.1016/j.nrleng.2020.08.002

[12] Gonzalez NR Amin-Hanjani S Bang OY Adult moyamoya disease and syndrome: current perspectives and future directions: a scientific statement from the American Heart Association/American Stroke Association. Stroke. 2023;54(10):e465-e479. 37609846 10.1161/STR.0000000000000443

[13] Kashiwazaki D Akioka N Kuwayama N Berlin grading system can stratify the onset and predict perioperative complications in adult moyamoya disease. Neurosurgery. 2017;81(6):986-991. 28605471 10.1093/neuros/nyx140

[14] Sahoo SS Suri A Bansal S Devarajan SLJ Sharma BS. Outcome of revascularization in moyamoya disease: evaluation of a new angiographic scoring system. Asian J Neurosurg. 2015;10(4):252-259. 26425151 10.4103/1793-5482.162681 PMC4558798

[15] Gao G Liu S-M Hao F-B Factors influencing collateral circulation formation after indirect revascularization for moyamoya disease: a narrative review. Transl Stroke Res. 2024;15(6):1005-1014. 37592190 10.1007/s12975-023-01185-x

[16] Rosi A Riordan CP Smith ER Scott RM Orbach DB. Clinical status and evolution in moyamoya: which angiographic findings correlate? Brain Commun. 2019;1(1):fcz029. 32954269 10.1093/braincomms/fcz029 PMC7425301

[17] Zhao X Wang C Ji Y Han C Wang M. Therapeutic effect of multiple burr hole operation combined with dural inversion and periosteal synangiosis for moyamoya disease. Br J Neurosurg. 2015;29(6):811-817. 26337649 10.3109/02688697.2015.1071318

[18] Kim T Oh CW Kwon OK Stroke prevention by direct revascularization for patients with adult-onset moyamoya disease presenting with ischemia. J Neurosurg. 2016;124(6):1788-1793. 26636391 10.3171/2015.6.JNS151105

[19] Kinugasa K Mandai S Kamata I Sugiu K Ohmoto T. Surgical treatment of moyamoya disease. Neurosurgery. 1993;32(4):527-531. 8474642 10.1227/00006123-199304000-00006

